# Bilateral Blindness in a Patient With Vestibular Schwannoma and Empty Sella Syndrome: A Case Report

**DOI:** 10.7759/cureus.101505

**Published:** 2026-01-14

**Authors:** Ammar M Bahati, Thamer H Alsharif, Mashhour A Alsuwat, Kemal Dizdarevic

**Affiliations:** 1 Department of Neurological Surgery, King Fahad General Hospital, Makkah, SAU; 2 Department of Medicine, Royal College of Surgeons in Ireland, Dublin, IRL; 3 Department of Neurosurgery, King Abdulaziz Specialist Hospital, Taif, SAU; 4 Department of Neurological Surgery, King Abdulaziz Specialist Hospital, Taif, SAU

**Keywords:** acoustic neuroma, bilateral blindness, empty sella syndrome, obstructive hydrocephalus, vestibular schwannoma, visual loss

## Abstract

We report a rare clinical presentation of a T4b vestibular schwannoma causing severe compression of the brainstem and fourth ventricle, associated with obstructive hydrocephalus and empty sella syndrome, in a patient presenting with profound neurological and ophthalmological deficits, most notably complete bilateral blindness. Radical microsurgical resection was achieved via a lateral suboccipital (retromastoid) approach, resulting in gross-total tumor removal with preservation of most cranial nerves. Postoperatively, the patient developed House-Brackmann grade III facial palsy. Hydrocephalus resolved without the need for cerebrospinal fluid diversion, gait instability improved, and visual deficits persisted. This case highlights the exceptional association of vestibular schwannoma with bilateral blindness and empty sella syndrome, emphasizing the importance of early recognition and timely surgical intervention in advanced tumors to minimize morbidity and optimize outcomes.

## Introduction

Vestibular schwannomas, also known as acoustic neuromas, are benign tumors arising from Schwann cells of the vestibular portion of the eighth cranial nerve [1,2]. They account for approximately 8-10% of all intracranial tumors and up to 80% of cerebellopontine angle (CPA) tumors [2]. Although advances in neuroimaging have improved early detection, large and advanced vestibular schwannomas remain clinically relevant because of their unpredictable growth patterns and significant mass effect on surrounding neurovascular structures [1,2].

According to the Hannover classification, T4b vestibular schwannomas represent the most advanced stage and are characterized by marked CPA extension with severe brainstem compression and deformation of the fourth ventricle, often resulting in obstructive hydrocephalus [3-5]. These tumors can cause displacement of multiple cranial nerves and significant neurological deterioration.

Typical clinical manifestations include progressive unilateral hearing loss, tinnitus, and imbalance, while facial nerve palsy is relatively uncommon at presentation [6]. However, atypical presentations may occur, particularly in advanced tumors complicated by hydrocephalus or chronic intracranial hypertension [7]. Neuro-ophthalmological manifestations, especially bilateral profound visual loss, are exceedingly rare [8]. The coexistence of empty sella syndrome, often associated with chronically elevated intracranial pressure, may further contribute to visual deterioration and complicate diagnosis and management [9].

We present a challenging case of a large T4b vestibular schwannoma associated with empty sella syndrome and complete bilateral blindness, highlighting the diagnostic considerations, surgical management, and postoperative outcome.

## Case presentation

A 26-year-old male presented with complete left-sided hearing loss, progressive gait instability, bilateral blindness, headache, and clinical signs of raised intracranial pressure. The visual loss had been gradually progressive over several months. There was no history suggestive of inflammatory, demyelinating, toxic, or hereditary optic neuropathy.

**Figure 1:**
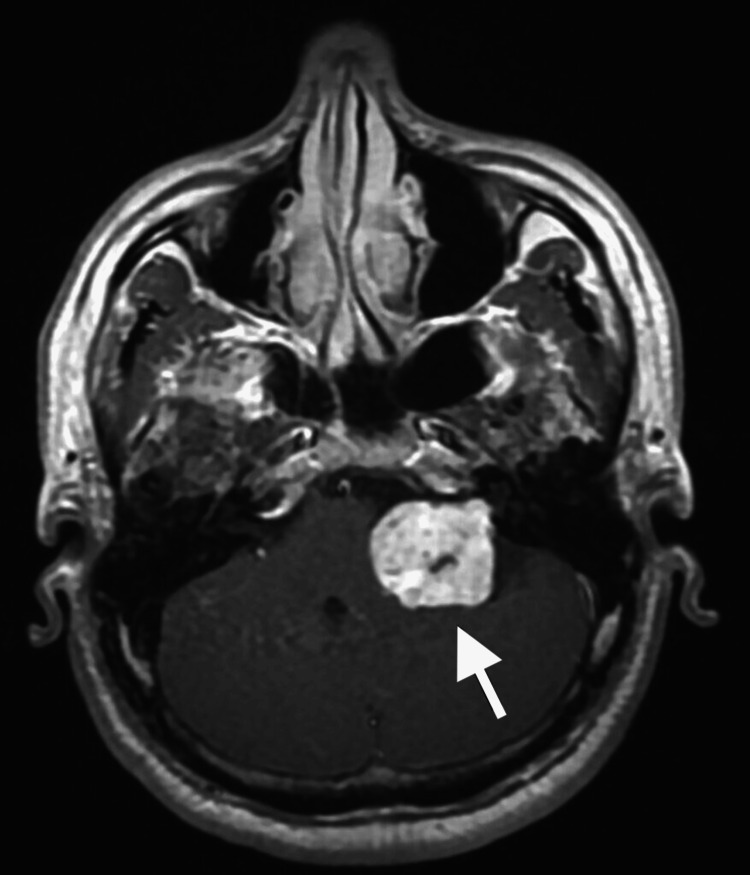
Preoperative MRI (T1-weighted with contrast) demonstrating a left-sided vestibular schwannoma measuring 38.2 × 31.3 mm (white arrow)

**Figure 2:**
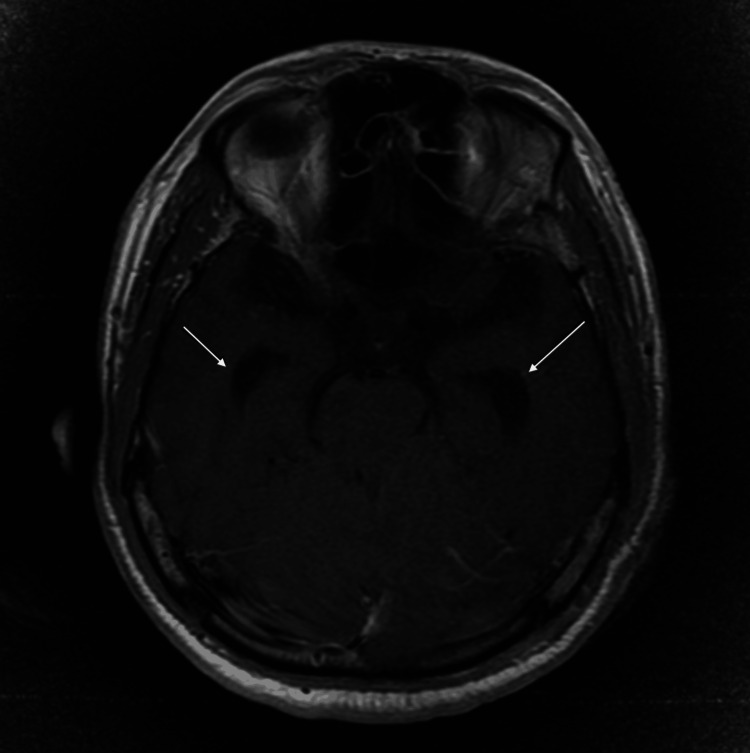
T1-weighted MRI demonstrating hydrocephalus (white arrows)

**Figure 3:**
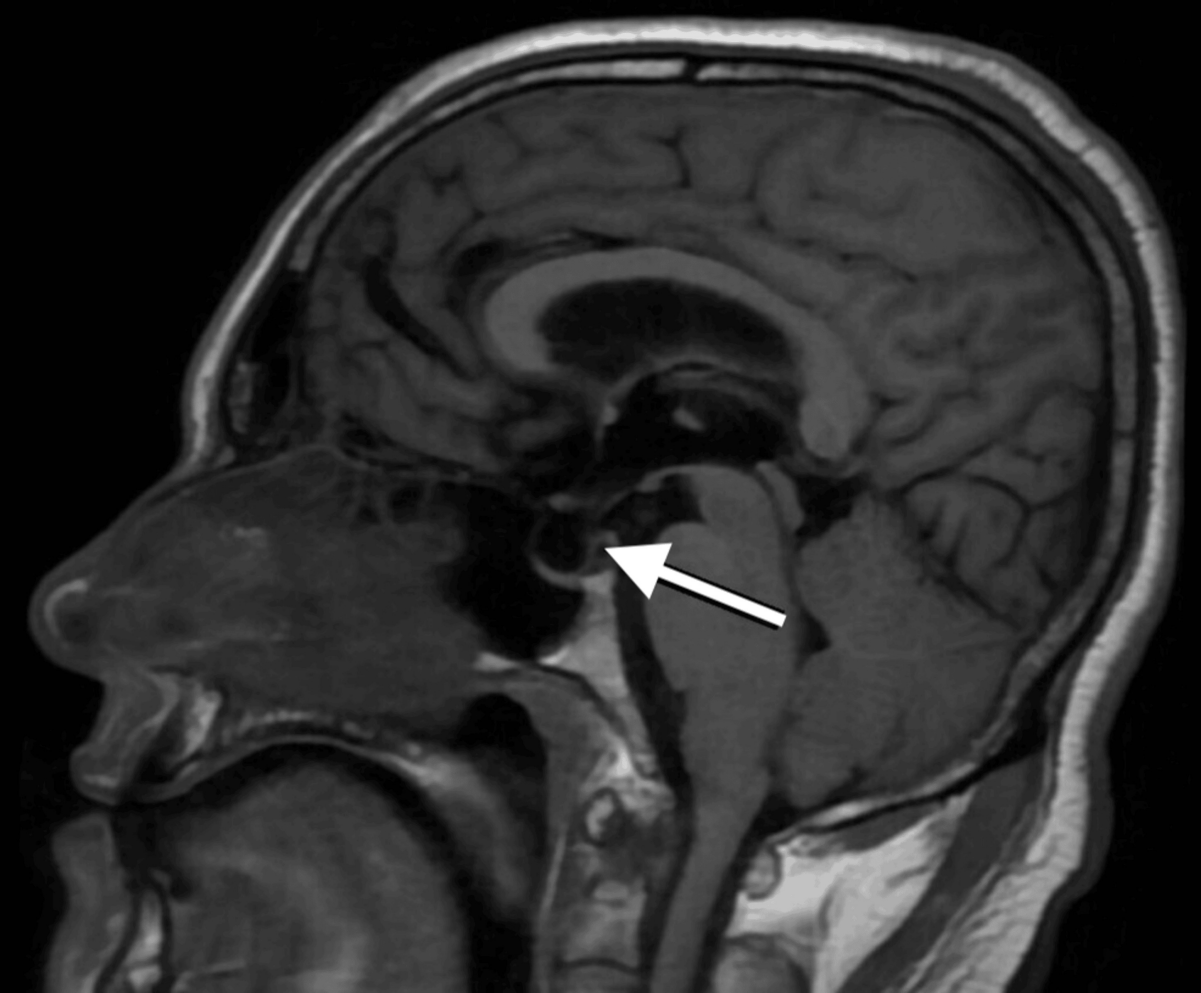
T1-weighted MRI demonstrating an empty sella (white arrow)

Magnetic resonance imaging (MRI) revealed a large left-sided vestibular schwannoma measuring 38.2 × 31.3 mm, classified as T4b according to the Hannover grading system, with severe compression of the brainstem and distortion of the fourth ventricle. Associated obstructive hydrocephalus and an empty sella were also noted (Figures 1-3) [3]. Ophthalmologic examination demonstrated bilateral optic disc pallor with absence of light perception in both eyes, consistent with chronic optic atrophy.

The patient underwent retrosigmoid craniotomy with complete tumor resection. He was positioned laterally with the head secured in a Mayfield three-pin head holder. A left retromastoid suboccipital approach was used, beginning with a curved incision behind the auricle, creation of one burr hole, and lateral suboccipital craniotomy. Bone drilling partially exposed the sigmoid and transverse sinuses. A reverse K-shaped dural incision allowed opening of the caudal CPA cistern and cerebrospinal fluid release, facilitating microsurgical dissection. The roof and lateral wall of the internal auditory canal were drilled to initiate tumor removal within the meatus, followed by debulking and mobilization of the caudal pole. Meticulous dissection of the capsule was performed from the lower, middle, and upper cranial nerve groups in the CPA. All cranial nerves were preserved, except for fibres of the VII/VIII complex, which were diffusely involved in the tumor.

**Figure 4:**
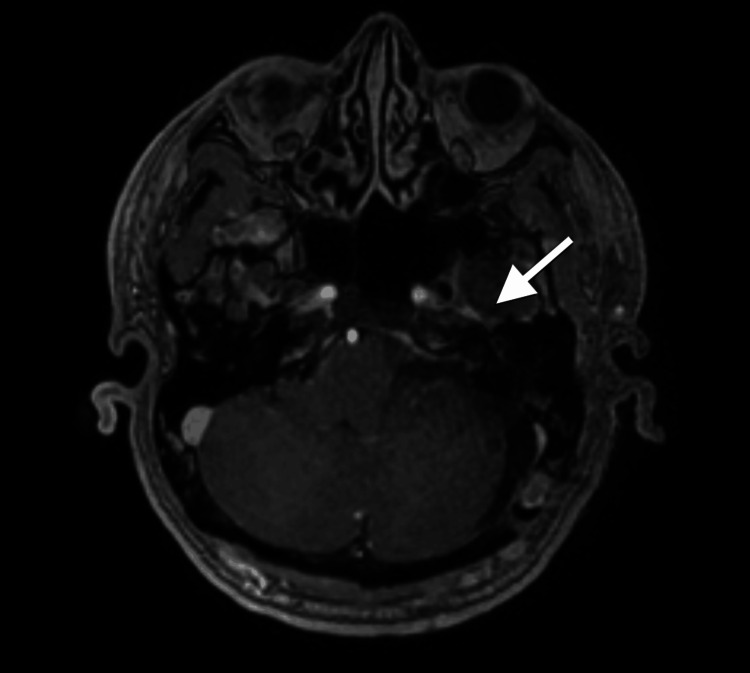
Postoperative MRI (T1-weighted with contrast) demonstrating complete tumor resection (white arrow)

The lesion consisted predominantly of firm, yellowish, vascularized tissue with smaller areas of softer, greyish consistency. A clear cleavage plane was identified between the tumor and surrounding structures, permitting safe radical excision while minimizing coagulation to preserve the facial nerve. No cerebrospinal fluid diversion was required intraoperatively. Postoperative MRI of the brain showed complete tumour resection and an empty sella (Figure 4). The patient was discharged on postoperative day 8. A new deficit of left facial paresis (House-Brackmann grade III, a widely accepted and non-proprietary clinical scale) was noted [10]. Gait instability improved, while visual deficits remained unchanged.

## Discussion

The management of T4B vestibular schwannomas is inherently complex, as these tumors are associated with a high risk of cranial nerve deficits and significant compression and extension to the brainstem, often requiring urgent surgical decompression to prevent further neurological deterioration [11].

Empty sella syndrome is commonly regarded as a radiological marker of chronic intracranial hypertension and, in some cases, may contribute to visual impairment and endocrine dysfunction through pituitary involvement [9].

Bilateral blindness in patients with vestibular schwannoma is exceptionally rare [8]. In the present case, the combination of chronic high intracranial pressure secondary to obstructive hydrocephalus, the anatomical and functional consequences of empty sella syndrome, and a delayed diagnosis offers a plausible explanation for the profound bilateral visual loss. The hydrocephalus was insidious, gradual, and obstructive in nature, resulting from severe compression and displacement of the fourth ventricle medially but without complete obliteration of the ventricle [11]. This displacement of the ventricle with significantly reduced but not obliterated CSF flow in the ventricle was probably the reason for the insidious course of hydrocephalus.

If the patient had developed acute hydrocephalus early due to complete ventricular obstruction in the presence of empty sella syndrome, the condition would likely have been recognized earlier at the primary care level, allowing treatment before the development of complete and irreversible blindness [12].

Careful postoperative monitoring for delayed disturbances in CSF circulation was therefore essential. Fortunately, gross-total resection of the tumor resulted in resolution of the hydrocephalus without the need for diversion procedures. Despite meticulous microsurgical technique and the anatomical preservation of facial cranial nerve fibers, the patient developed postoperative facial palsy. This outcome is not unexpected in cases of T4B vestibular schwannomas, given the advanced tumor stage and the extensive involvement of the CPA, even under strict intraoperative monitoring and nerve-sparing strategies [13,14].

## Conclusions

This case highlights the diagnostic and surgical challenges of large T4B vestibular schwannomas, particularly when associated with empty sella syndrome and severe visual impairment. Bilateral blindness in this setting is exceptionally rare and is most likely related to long-standing intracranial hypertension from obstructive hydrocephalus rather than a direct effect of the tumor itself. Early recognition and timely intervention remain critical to reduce neurological morbidity. Despite significant preoperative deficits, radical microsurgical resection can be safely achieved, resulting in effective brainstem decompression and resolution of hydrocephalus. This report underscores the importance of individualized surgical planning, careful interpretation of atypical presentations, and acknowledgment of diagnostic limitations in the management of advanced vestibular schwannomas.
